# Sec15 links bud site selection to polarised cell growth and exocytosis in *Candida albicans*

**DOI:** 10.1038/srep26464

**Published:** 2016-05-26

**Authors:** Pan Pan Guo, Jie Ying Au Yong, Yan Ming Wang, Chang Run Li

**Affiliations:** 1 Institute of Health Sciences, Anhui University, Hefei 230601, China; 2 Institute of Molecular and Cell Biology, A*STAR, 61 Biopolis Drive, 138673 Singapore

## Abstract

The exocyst plays a crucial role in the targeting of secretory vesicles to the plasma membrane during exocytosis. It has been shown to be involved in diverse cellular processes including yeast budding. However, the mechanism of the exocyst regulating yeast budding has not been fully elucidated. Here we report a novel interaction between the exocyst component Sec15 and the Ras-family GTPase Rsr1, a master regulator of bud-site-selection system, in the fungus *Candida albicans*. We present several lines of evidence indicating physical and genetic interaction of Sec15 with Rsr1. *In vitro* binding assays and co-immunoprecipitation studies showed that Sec15 associated physically with Rsr1. Deletion of *RSR1* completely abolished the polarised localisation of Sec15 as well as all the other exocyst components in both yeast and hyphal cells, suggesting a functional interaction between Sec15 and Rsr1. We also show that *C. albicans* Sec15 interacts directly with the polarity determinant Bem1 and the type V myosin, Myo2. Disruption of the interaction by shutting off *SEC15* results in mislocaliztion of Bem1-GFP. These findings highlight the important role of Sec15 in polarised cell growth by providing a direct functional link between bud-site-selection and exocytosis.

Exocytosis is a fundamental membrane trafficking event in eukaryotic cells in which membrane proteins or lipids are incorporated into the plasma membrane and vesicle contents are secreted to the exterior of the cell. It is essential for cells to absorb nutrients, communicate with the environment and each other, generate and maintain functional shapes^1^. The precise temporal and spatial regulation of polarised exocytosis is important for diverse biological events such as the establishment of a new bud in yeast^2^,^3^, epithelial cell polarisation^4^ and neuron development^5^. Polarised exocytosis takes place in several steps, which are evolutionarily conserved in eukaryotes. First, post-Golgi vesicles are transported by the type V myosin, Myo2, along polarised actin cables to sites of cell surface growth. Second, the vesicles are tethered at specific sites of the plasma membrane^6^,^7^. Finally, the vesicle membrane fuses with the plasma membrane in a process mediated by interactions between the SNARE family proteins located both on the vesicles and at the target membrane^8^.

The tethering of the secretory vesicles at the plasma membrane is mediated by the exocyst^9^,^10^. The exocyst is an evolutionarily conserved protein complex composed of Sec3, Sec5, Sec6, Sec8, Sec10, Sec15, Exo70 and Exo84. The exocyst components are localised at the bud tip of small-budded cells or mother-daughter cell junction (bud neck) of large-budded cells, the sites of plasma membrane where active exocytosis and membrane expansion take place^11^,^12^,^13^,^14^. The exocyst has been shown to be involved in diverse cellular processes requiring polarised exocytosis such as yeast budding, epithelial polarity establishment, neurite outgrowth, and ciliogenesis^15^,^16^,^17^,^18^. However, the mechanism of the exocyst regulating yeast budding has not been fully elucidated.

*C. albicans* is a multimorphic opportunistic human fungal pathogen^19^. In recent years, the role of exocyst components in the morphogenesis especially in the hyphal morphogenesis has been studied extensively in *C. albicans*^20^,^21^. Most recently, *C. albicans* Sec15 and Sec6 have been shown to play an important role in the delivery of cell wall components and hyphal branching^22^,^23^. In the budding yeast *Saccharomyces cerevisiae*, Sec15 has been shown to interact genetically as well as physically with activated Sec4, one of the founding members of the small rab GTPase family and a master regulator of post-Golgi vesicle trafficking^14^,^24^. Sec15 has also been shown to interact with the polarity establishment component Bem1 in yeast^25^. In *Drosophila*, Sec15 has been identified as a synthetic suppressor of oncogenic Ras by interacting with Eiger/TNF^26^. Here we show that *C. albicans* Sec15 interacts both physically and genetically with the Ras-family GTPase Rsr1, a master regulator of the bud site selection system, thus providing a direct link between the bud site selection system and exocytosis. We also show that Sec15 directly associates with the polarity determinant Bem1 and the unconventional myosin motor myosin V, Myo2. Therefore, Sec15 may play a central role in the polarised growth and exocytosis in *C. albicans*.

## Results

### Sec15 is required for normal cell growth and the maintenance of hyphal extension

**Figure 1:**
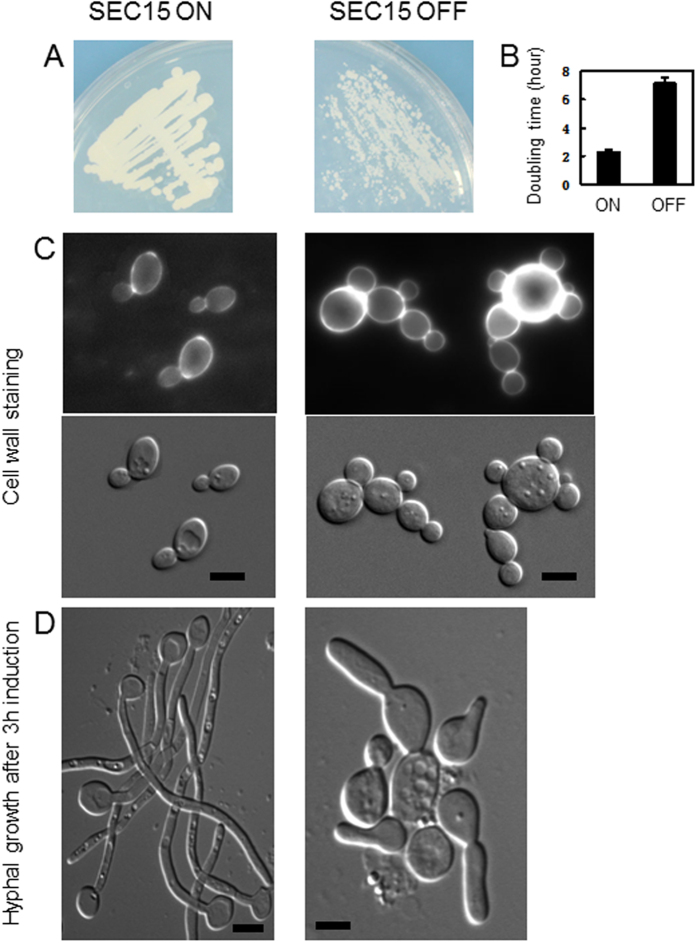
*C. albicans sec15* shut off mutant cells showed morphological defect. (**A**) *SEC15* shut off strain (strain AHU2) was first grown at 30 °C in maltose medium overnight, then diluted and equally inoculated onto maltose or glucose agar medium and incubated at 30 °C for 3 days. (**B**) The same overnight culture was diluted into liquid maltose or glucose medium and grown at 30 °C with vigorous shaking. Growth was monitored by measuring OD600 at intervals. Doubling times were calculated from the average OD values during the exponential phase of growth from three independent experiments. Data were means ± SD from three independent experiments. (**C**) *SEC15* shut off strain (strain AHU2) was first grown at 30 °C in maltose and glucose medium overnight respectively. The overnight cultures were diluted into fresh liquid maltose or glucose medium and grown at 30 °C for further 3 hours. An aliquot of cells was then stained by Calcofluor White. Bars, 5 μm. (**D**) The same overnight cultures as above were diluted into liquid maltose or glucose medium plus 10% serum and grown at 37 °C for 3 hours. Bars, 5 μm.

Because *SEC15* is essential for viability in *S. cerevisiae*, we generated a *C. albicans SEC15* conditional mutant by deleting one copy of *SEC15* and placing the other copy under the control of *MAL2* promoter which can be shut off when grown in medium containing glucose. Strain construction was verified by colony PCR. The *sec15* shut off cells grown under repressive conditions showed slow growth and significant morphological defects compared with the *SEC15* on cells grown under permissive conditions. First, the *sec15* shut off cells formed much smaller colonies than the *SEC15* on cells after 3 days of growth on agar medium (Fig. 1A). Furthermore, the doubling time of the *sec15* shut off cells was almost 3 times longer of the doubling time of the *SEC15* on cells (Fig. 1B). These results indicate that the *sec15* shut off cells grew much more slowly than the *SEC15* on cells. Second, the *sec15* shut off cells were much larger than the *SEC15* on cells. Cell wall staining with Calcofluor White revealed that the *sec15* shut off cells showed random budding pattern in contrast to the bipolar budding pattern of the *SEC15* on cells (Fig. 1C). Furthermore, the *sec15* shut off cells showed cell separation defect (Fig. 1C). Third, the *sec15* shut off cells showed severe hyphal growth defects. More than 20 percent of the *sec15* shut off cells were not able to generate germ tubes under hyphal inducing condition (100 cells were scored each for three repeats). While most of the *sec15* shut off cells were able to generate a germ tube during the initial phase of hyphal development, they were not able to maintain the hyphal extension in prolonged growth. As a result, the *sec15* shut off cells produced much shorter hyphae than the *SEC15* on cells (Fig. 1D).

**Figure 2:**
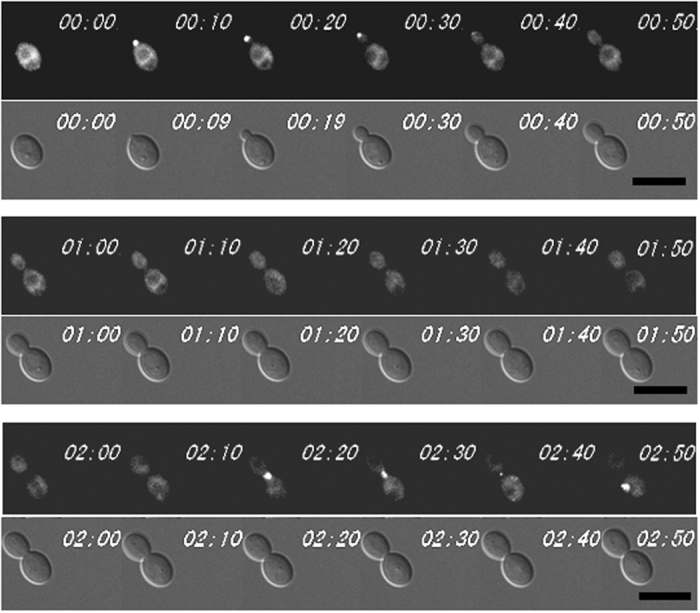
Sec15-GFP shows cell cycle-dependent localisation in *C. albicans*. Cells expressing Sec15-GFP (strain WYL41) were examined by time-lapse microscopy at intervals of 10 mins. Bars, 5 μm.

To get more information about the function of *SEC15*, we then examined the localisation of Sec15-GFP. The Sec15-GFP fusion protein was fully functional as reported previously^27^. Results by time-lapse microscopy showed that Sec15-GFP localisation could only be observed in two phases of the cell cycle: it localised to bud tips in the small-budded cells and at the bud neck in cells undergoing cytokinesis (Fig. 2). These results are consistent with the typical localisation pattern of exocyst proteins in *S. cerevisiae*^1^,^7^.

### Sec15 interacts with Rsr1-GDP *in vitro* and *in vivo*

**Figure 3:**
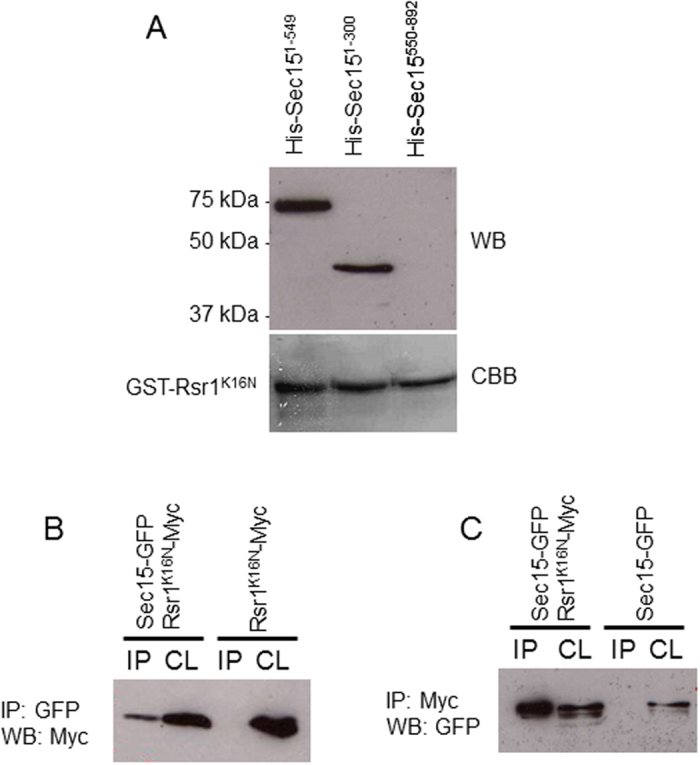
Sec15 binds to Rsr1-GDP *in vitro* and *in vivo*. (**A**) Recombinant GST-Rsr1^K16N^ purified from *E. coli* was pre-immobilized on glutathione beads and mixed with equal amounts of 20 μg of purified His6-Sec15^1–549^, His6-Sec15^1–300^ and His6-Sec15^550–892^. Bound GST fusion proteins were detected by Coomassie brilliant blue (CBB) staining and associated His6-tagged proteins were detected with anti-His antibody. (**B**) Cell lysates (CL) were prepared from *C. albicans* cells co-expressing Sec15-GFP and Rsr1^K16N^-Myc or Rsr1^K16N^-Myc alone, subjected to anti-GFP immunoprecipitation (IP) followed by anti-Myc western blot (WB). (**C**) Cell lysates were prepared from *C. albicans* cells co-expressing Sec15-GFP and Rsr1^K16N^-Myc or Sec15-GFP alone, subjected to anti-Myc immunoprecipitation followed by anti-GFP western blot.

An interaction between Rsr1-GDP (but not Rsr1-GTP) and Sec15 has been detected by a large scale two-hybrid study in *S. cerevisiae*^28^. To verify whether there is any physical interaction between Rsr1-GDP and Sec15 in *C. albicans*, *in vitro* binding assays were carried out. To this end, a recombinant Rsr1 GDP-locked form GST-Rsr1^K16N^ was constructed and purified from *E. coli* by glutathione-Sepharose affinity chromatography. Indeed, GST-Rsr1^K16N^ was found to bind with the N-terminal regions of Sec15, His-Sec15^1–300^ (amino acids 1 to 300, expected size about 45 kDa) and His-Sec15^1–549^ (amino acids 1 to 549, expected size about 74 kDa)(Fig. 3A). In contrast, the C-terminal region His-Sec15^550–892^ (amino acids 550 to 892) did not bind with GST-Rsr1^K16N^. These results indicate that Rsr1-GDP physically interacts with the N-terminal region of Sec15 *in vitro*.

To further verify whether Rsr1-GDP interacts with Sec15 *in vivo*, a *C. albicans* strain co-expressing Sec15-GFP and Rsr1^K16N^-Myc and strains expressing Sec15-GFP or Rsr1^K16N^-Myc alone were constructed respectively. When Sec15-GFP was pulled down from the cell lysate using anti-GFP antibody, Rsr1^K16N^-Myc was detected in the precipitate of the cells co-expressing Sec15-GFP and Rsr1^K16N^-Myc but was not detected in the precipitate of the cells expressing Rsr1^K16N^-Myc alone (Fig. 3B). Reciprocally, when Rsr1^K16N^-Myc was pulled down from the cell lysate using anti-Myc antibody, Sec15-GFP was detected in the precipitate of the cells co-expressing Sec15-GFP and Rsr1^K16N^-Myc but was not detected in the precipitate of the cells expressing Sec15-GFP alone. These results demonstrate physical association of Rsr1-GDP with Sec15 *in vivo* in *C. albicans*.

### SEC15 genetically interacts with RSR1

**Figure 4:**
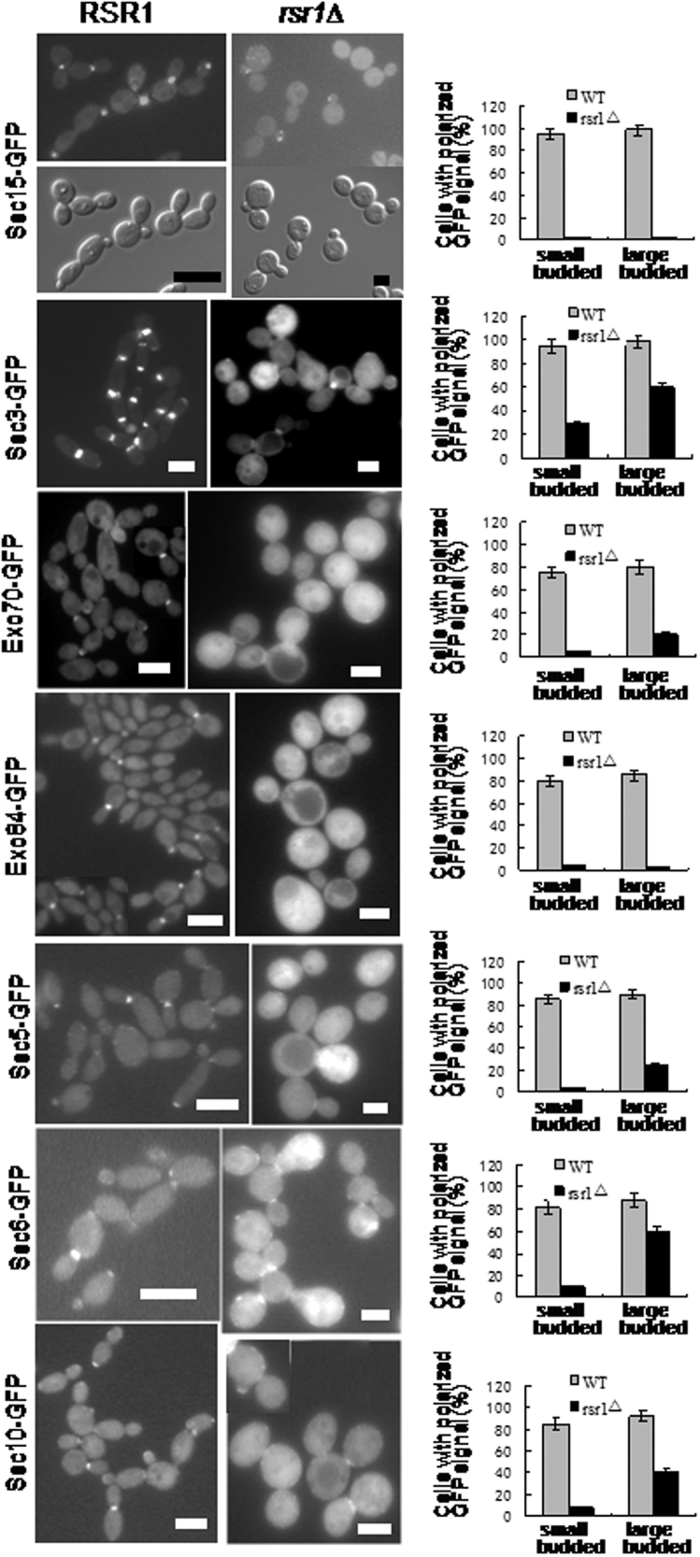
Exocyst subunits showed localisation defects in *rsr1*Δ cells. Left panel, cells expressing exocyst-GFP in wild type background and *rsr1*Δ mutant background were grown to early log phase in GMM at 30 °C. Cells were harvested and examined by fluorescence microscopy. Bars, 5 μm. Right panel, the percentage of cells with polarised GFP signal in small-budded and medium/large-budded cells was quantified. Error bars indicate SD for three separate experiments. For each experiment, 200–300 cells were scored for each strain.

Taken together, the above results indicate that Sec15 interacts with Rsr1-GDP both *in vitro* and *in vivo*. To determine whether there is any genetic interaction between *RSR1* and *SEC15*, Sec15-GFP localisation was examined in the *rsr1*Δ null mutant. In a wild type background, Sec15-GFP localises to the bud tip of small-budded cells and the bud neck of large-budded cells (Fig. 2). However, Sec15-GFP signal was not observed either at the bud tip or the bud neck in the *rsr1*Δ null mutant cells (Fig. 4, top). Instead, randomly localised Sec15-GFP spots were observed at the cell cortex in the small bud in a fraction of *rsr1*Δ null mutant cells. These results indicate that the polarised localisation of Sec15-GFP was completely abolished in the *rsr1*Δ mutant cells. These results indicate that the polarised localisation of Sec15-GFP depends on Rsr1. Taken together, both the functional and physical interactions between Sec15 and Rsr1 strongly support the idea that Rsr1 plays an important role in determining the localisation of the exocyst proteins to the site of bud growth.

To determine whether other exocyst components also mislocalise in the *rsr1*Δ mutant cells, the localisation of all the other exocyst components except Sec8 (whose GFP-tagging was not successful) was examined. All the GFP fusion proteins were functional as shown previously^21^. In a wild type background, all these exocyst components showed similar localisation pattern to Sec15, i.e. they localise to the bud tips of small-budded cells and the bud-neck of large-budded cells undergoing cytokinesis (Fig. 4). However, all these exocyst components showed localisation defects in the *rsr1*Δ mutant cells, although to a lesser extent compared with Sec15. Similar to Sec15, the bud tip localisation in the small-budded cells was almost completely abolished for all the exocyst components except Sec3, which still showed bud tip localisation in about 30% of the *rsr1*Δ mutant cells. Contrary to Sec15, the bud neck localisation in the large-budded cells could be observed in more than 20% of the *rsr1*Δ null mutant cells for all the other exocyst components except Exo84, which showed no bud neck localisation in the *rsr1*Δ mutant cells. However, compared with wild type background cells, the bud neck localisation for all the exocyst components showed significant defects in the *rsr1*Δ mutant cells in the following two aspects: first, the number of the *rsr1*Δ mutant cells showing bud neck localisation was much less than that of wild type background cells; second, the GFP signal at the bud neck in the *rsr1*Δ mutant cells was much weaker than that in the wild type background cells.

**Figure 5:**
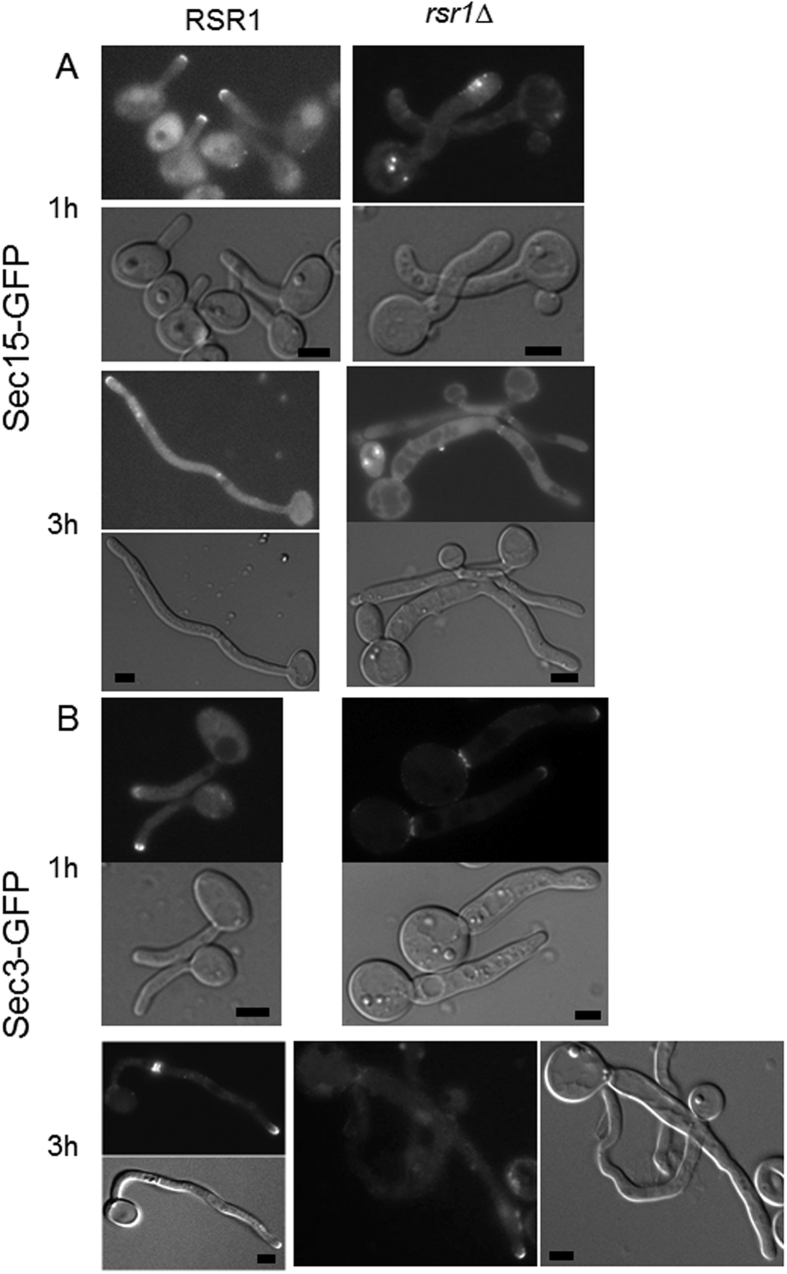
Sec15-GFP was mislocalised in *rsr1*Δ mutant hyphae. (**A**) cells expressing Sec15-GFP in wild type and *rsr1*Δ mutant were grown overnight in GMM at 30 °C. Cells were then diluted into fresh GMM medium plus 10% serum and incubated at 37 °C for the indicated time period. Bars, 5 μm. (**B**) cells expressing Sec3-GFP in wild type and *rsr1*Δ mutant were grown overnight in GMM at 30 °C. Cells were then diluted into fresh GMM medium plus 10% serum and incubated at 37 °C for the indicated time period. Bars, 5 μm.

Next we examined the exocyst localisation under hyphal growth condition. In a wild type background, Sec15-GFP localised to the hyphal tip at 1 h of hyphal induction (Fig. 5A). At 3 h of hyphal induction, both hyphal tip and septum localisation of Sec15-GFP could be observed. In contrast, hyphal tip localisation of Sec15-GFP was completely abolished in *rsr1*Δ mutant cells at both 1 h and 3 h of hyphal induction, although septum localisation of Sec15-GFP could be observed at 3 h of hyphal induction. Similar localisation pattern was observed for all the other exocyst subunits except Sec3 (Fig. 5B and supplemental Fig. S1–5). Contrary to Sec15-GFP, hyphal tip localisation of Sec3-GFP could clearly be observed in *rsr1*Δ mutant cells at both 1 h and 3 h of hyphal induction.

To summarize, all the exocyst components examined showed severe localisation defects in the *rsr1*Δ mutant cells. First, the bud tip and bud neck localisation of Sec15 and Exo84 was completely abolished in the *rsr1*Δ yeast cells. Second, the bud tip localisation of Sec3, Sec5, Sec6, Sec10 and Exo70 was abolished in most of the *rsr1*Δ yeast cells while the bud neck localisation of Sec3, Sec5, Sec6, Sec10 and Exo70 was abolished in more than 40% of the *rsr1*Δ yeast cells. Third, Sec3, Sec5, Sec6, Exo70 and Exo84 mislocalised to cytoplasm in most of the *rsr1*Δ yeast cells. Fourth, the hyphal tip localisation of all the exocyst subunits except Sec3 was abolished in the *rsr1*Δ cells under hyphal inducing condition. These results support the idea that Rsr1 plays a role in the positioning of the exocyst complex.

### Sec15 interacts with the polarity determinant Bem1

**Figure 6:**
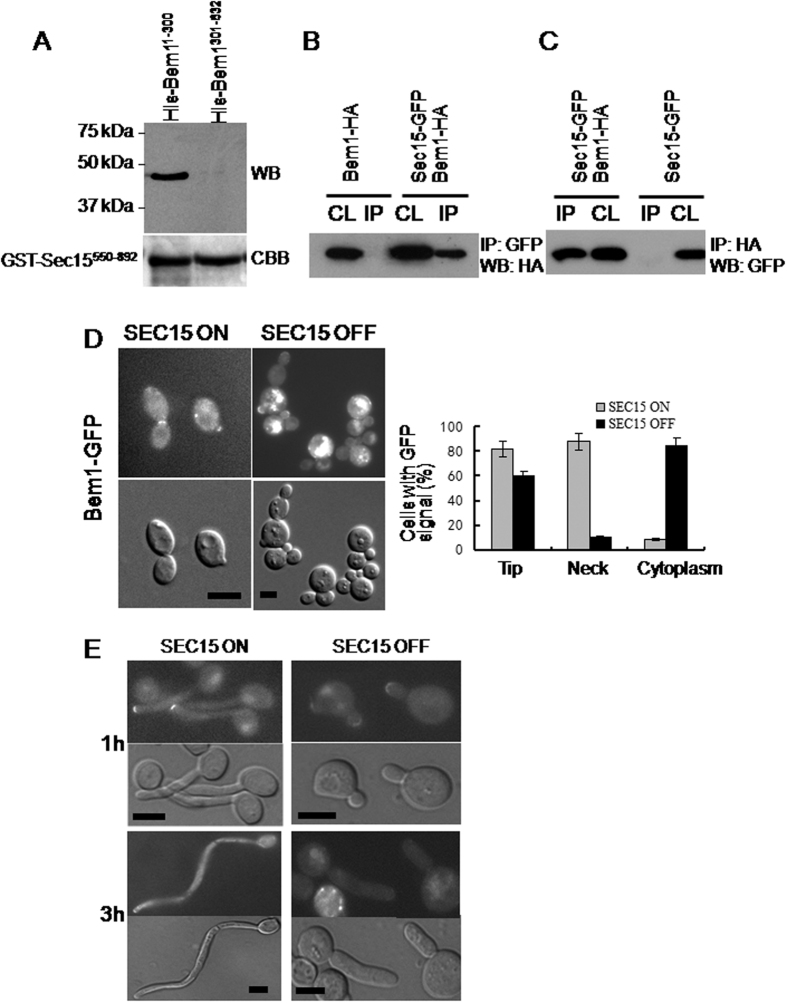
Sec15 binds to Bem1 *in vitro* and *in vivo*. (**A**) Recombinant GST-Sec15 C-terminal (amino acid 550–892) purified from *E. coli* was pre-immobilized on glutathione beads and mixed with equal amounts of 20 μg of purified His6-Bem1^1–300^, and His6-Bem1^301–632^. Bound GST fusion proteins were detected by CBB staining and associated His6-tagged proteins were detected with anti-His antibody. (**B**) Cell lysates (CL) were prepared from *C. albicans* cells co-expressing Sec15p-GFP and Bem1-HA or Bem1-HA alone, subjected to anti-GFP immunoprecipitation (IP) followed by anti-HA western blot (WB). (**C**) Cell lysates were prepared from *C. albicans* cells co-expressing Sec15-GFP and Bem1-HA or Sec15p-GFP alone, subjected to anti-HA immunoprecipitation followed by anti-GFP western blot. (**D**) Left panel, Bem1-GFP localisation was examined in *SEC15* shut off strain (AHU19) after 3h of re-culture in maltose or glucose medium diluted from overnight culture. Bars, 5 μm. Right panel, the percentage of cells with polarised GFP signal in small-budded and medium/large-budded cells was quantified. Error bars indicate SD for three separate experiments. For each experiment, 200–300 cells were scored for each strain. Bars, 5 μm. (**E**) Bem1-GFP localisation was examined in *SEC15* shut off strain under hyphal growth condition. Bars, 5 μm.

A direct physical interaction between Sec15 and Bem1 has been demonstrated in *S. cerevisiae*^25^. To determine whether similar interaction exists in *C. albicans*, *in vitro* binding assays between the C-terminal 343 amino acids of Sec15 and Bem1 was performed. Indeed, GST-Sec15^550–892^ was found to bind with the N-terminal 300 amino acids of Bem1, His-Bem1^1–300^ (expected size 43.5 kDa). In contrast, GST-Sec15^550–892^ did not bind with the C-terminal region of Bem1, His-Bem1^301–632^ (Fig. 6A). These results are consistent with the results in *S. cerevisiae*^25^. Therefore, *C. albicans* Sec15 associates with Bem1 *in vitro*.

To verify whether Sec15 interacts with Bem1 *in vivo*, a *C. albicans* strain co-expressing Sec15-GFP and Bem1-HA and strains expressing Sec15-GFP or Bem1-HA alone were constructed respectively. When Sec15-GFP was pulled down from the cell lysate using anti-GFP antibody, Bem1-HA was detected in the precipitate of the cells co-expressing Sec15-GFP and Bem1-HA but was not detected in the precipitate of the cells expressing Bem1-HA alone (Fig. 6B). Reciprocally, when Bem1-HA was pulled down from the cell lysate using anti-HA antibody, Sec15-GFP was detected in the precipitate of the cells co-expressing Sec15-GFP and Bem1-HA but was not detected in the precipitate of the cells expressing Sec15-GFP alone (Fig. 6C). These results are consistent with physical interaction of Sec15 with Bem1 *in vivo* in *C. albicans*.

To determine whether there is any genetic interaction between Sec15 and Bem1, Bem1-GFP localisation was examined in the *SEC15* shut off strain. When *SEC15* was on, Bem1-GFP localised to the bud tips of small-budded cells and the bud-neck of large-budded cells undergoing cytokinesis as in wild type cells (Fig. 6D). These results are consistent with Bem1 localisation in *S. cerevisiae*^25^. However, when *SEC15* was shut off, the bud neck localisation of Bem1-GFP was lost in most of the large-budded cells although most of the small-budded cells still showed bud tip localisation of Bem1-GFP (Fig. 6D). Furthermore, Bem1-GFP mislocalised to the cytoplasm of the mother cells in most of the *SEC15* shut off cells.

Under hyphal inducing conditions, Bem1-GFP localised to the hyphal tip when *SEC15* was on (Fig. 6E, left panel). When *SEC15* was shut off, hyphal tip localisation of Bem1-GFP could be observed at 1 h of hyphal induction. However, hyphal tip localisation of Bem1-GFP was completely abolished in *SEC15* shut off cells at 3 h of hyphal induction (Fig. 6E, right panel). These results clearly show a severe localisation defect for Bem1-GFP in *SEC15* shut off cells, indicating a strong genetic interaction between Sec15 and Bem1 in *C. albicans*.

### Sec15 interacts with Myo2 both *in vitro* and *in vivo*

**Figure 7:**
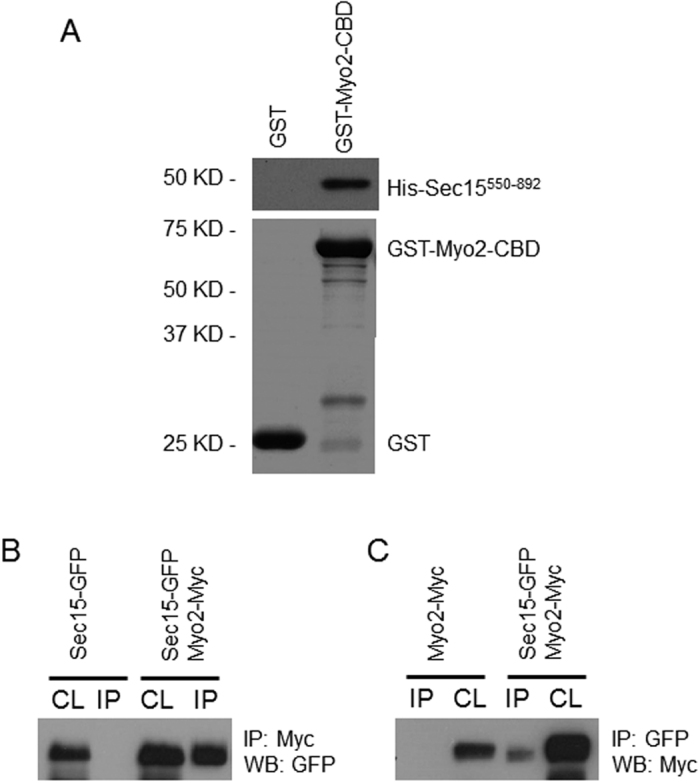
Sec15 binds to Myo2 *in vitro* and *in vivo*. (**A**) Recombinant GST-Myo2-CBD or GST alone purified from *E. coli* was pre-immobilized on glutathione beads and mixed with equal amounts of 20 μg of purified His6-Sec15^550–892^. Bound GST fusion proteins were detected by CBB staining and associated His6-tagged proteins were detected with anti-His antibody. (**B**) Cell lysates (CL) were prepared from *C. albicans* cells co-expressing Sec15-GFP and Myo2-Myc or Sec15-GFP alone, subjected to anti-Myc IP followed by anti-GFP western blot. (**C**) Cell lysates were prepared from *C. albicans* cells co-expressing Sec15-GFP and Myo2-Myc or Myo2-myc alone, subjected to anti-GFP IP followed by anti-Myc western blot.

It has been reported that Sec15 interacts directly with Myo2 in *S. cerevisiae*^29^. To determine whether similar interaction exists in *C. albicans*, *in vitro* binding assay between the C-terminal 343 amino acids of Sec15 (His-Sec15^550–892^) and the cargo-binding domain (CBD) of Myo2 was performed. Indeed, recombinant GST-Myo2-CBD strongly pulled down recombinant His-Sec15^550–892^ (Fig. 7A). In contrast, recombinant GST only did not pull down His-Sec15^550–892^. These results indicate that the C-terminal of Sec15 interacts directly with the CBD of Myo2 *in vitro*. This result is consistent with the result reported in *S. cerevisiae*^29^.

To test whether Sec15 interacts with Myo2 *in vivo*, a *C. albicans* strain co-expressing Sec15-GFP and Myo2-Myc and strains expressing Sec15-GFP or Myo2-Myc alone were constructed respectively. When Sec15-GFP was pulled down from the cell lysate using anti-GFP antibody, Myo2-Myc was detected in the precipitate of the cells co-expressing Sec15-GFP and Myo2-Myc but was not detected in the precipitate of the cells expressing Myo2-Myc alone (Fig. 7B). Reciprocally, when Myo2-Myc was pulled down from the cell lysate using anti-Myc antibody, Sec15-GFP was detected in the precipitate of the cells co-expressing Sec15-GFP and Myo2-Myc but was not detected in the precipitate of the cells expressing Sec15-GFP alone (Fig. 7C). These results indicate that Sec15 physically interacted with Myo2 *in vivo* in *C. albicans*.

## Discussion

The exocyst plays a crucial role in the targeting of secretory vesicles to the plasma membrane during exocytosis. It has been shown to be involved in diverse cellular processes including yeast budding^2^,^3^. However, the molecular mechanism of exocyst regulating yeast budding remains unknown. Here we present several lines of evidence supporting a direct interaction between the exocyst component Sec15 and bud site selection determinant Rsr1 in *C. albicans*. First, *in vitro* binding assays with recombinant Rsr1^K16N^ and fragments of Sec15 indicate that the N-terminal region of Sec15 directly binds to Rsr1^K16N^ *in vitro*. Second, co-immunoprecipitation experiments with *C. albicans* cells co-expressing Sec15-GFP and Myc-Rsr1^K16N^ indicate that Sec15-GFP associates with Myc-Rsr1^K16N^ *in vivo*. Third, a complete loss of polarised localisation of Sec15-GFP was observed in *rsr1*Δ null mutant cells under both yeast and hyphal growth conditions. In contrast, polarised localisation of polarity establishment protein Bem1 was observed in both germ tubes and mature hyphae of *rsr1*Δ null mutant cells^30^. These results indicate that the loss of polarised localisation in *rsr1*Δ null mutant is specific to the exocyst. Last, the *SEC15* shut off cells showed a random budding pattern in contrast to the bipolar budding pattern of wild type cells. The fact that another exocyst component, the *sec3*Δ null mutant cells showed normal bipolar budding pattern^27^ suggests that the random budding pattern is specific to *SEC15* shut off cells. Taken together, these results strongly support that Sec15 interacts with Rsr1 both physically and genetically. Rsr1 is a master regulator of the bud-site-selection system in *S. cerevisiae*^31^. Thus the direct interaction between Sec15 and Rsr1 indicates that Sec15 may provide an important functional link between the bud-site-selection system and the secretory machinery.

Furthermore, several lines of evidence have been obtained in this study indicating that Sec15 directly associates with the polarity determinant Bem1 in *C. albicans*. Similar results have been reported in the budding yeast *S. cerevisiae*^25^. Bem1 is a scaffold protein that is required to maintain the Cdc42 module at the incipient budding site and is therefore essential for the proper function of the Cdc42 signaling cascade which leads to the initiation and stabilization of cell polarity in *S. cerevisiae*^32^. The interaction between Sec15 and Bem1 may provide a tight functional link between the secretory machinery and the polarity establishment machinery. Another functional link between the secretory machinery and the polarity establishment machinery has been suggested by the detection of association between the exocyst subunit Sec3 and Cdc42 in *S. cerevisiae*^33^. Our previous study has shown that GFP-Cdc42 showed no significant localisation defect in *sec3*Δ null mutant cells under normal growth temperature of 30 °C in *C. albicans*^27^, while Bem1-GFP showed severe localisation defect in the *SEC15* shut off cells under similar growth conditions (Fig. 6D). These results indicate that Sec15, rather than Sec3, might play a major role in linking the secretory machinery and the polarity establishment machinery in *C. albicans*.

A recent study of the role of Sec15 in *C. albicans* hyphal formation by Chavez-Dozal *et al.*^23^ shows that the Spitzenkörper marker Mlc1-GFP, polarisome Spa2-GFP and exocyst subunit Exo70-GFP are mislocalised in strain tetR-*SEC15* under repressive conditions. Consistent with these results, polarity establishment protein Bem1 is mislocalised in the *MAL2*-*SEC15* conditional mutant hyphae grown under repressive conditions (Fig. 6E). It is noteworthy that Chavez-Dozal and co-workers used the more strictly regulated TetR promoter, other than the leakier *MAL2* promoter, for conditional expression of *SEC15* and showed that *SEC15* was absolutely required for viability (cell death occurred within 5 hours of *SEC15* shut off). Interestingly, all of Mlc1-GFP, Spa2-GFP and Exo70-GFP show no localisation defect in strain tetR-SEC6 under repressive conditions^23^. These results further support that Sec15 might play a critical role in linking the secretory machinery and the polarity establishment machinery in *C. albicans*.

In yeast *S. cerevisiae*, the unconventional myosin motor myosin V, Myo2, transports secretory vesicles to sites of exocytosis^34^. The C-terminal cargo-binding domain of Myo2 plays a critical role in the attachment of Myo2 to cargoes. In this study we find that the C-terminal region of Sec15 binds directly to the CBD of Myo2 in *C. albicans*, consistent with a previous study in yeast^29^. Yeast Sec15 also binds to the rab GTPase Sec4, providing a physical link between activated Sec4 on the vesicle surface and the other subunits of the exocyst complex^14^. Therefore, Sec15 plays a crucial role both in the recruitment of exocyst complex to the surface of secretory vesicles through interaction with Sec4 and in the transport of exocyst/secretory vesicles to sites of exocytosis by direct interaction with Myo2.

**Figure 8:**
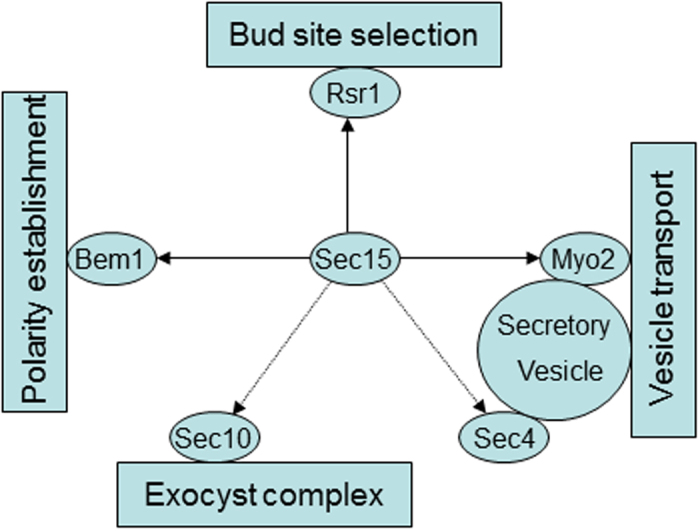
Schematic representation of Sec15 interactions. Interactions identified in both *S. cerevisiae* and *C. albicans* are indicated by solid lines. Interactions identified only in *S. cerevisiae* are indicated by dashed lines.

Taken together, Sec15 provides multiple functional links via interaction with various proteins (Fig. 8). First, Sec15 provides a functional link between the bud-site-selection system and the secretory machinery by interacting with the Ras-family GTPase Rsr1. Second, Sec15 provides a functional link between the polarity establishment machinery and the secretory machinery by interacting with polarity determinant Bem1. Third, Sec15 provides a functional link between secretory vesicles and the exocyst complex by interacting with type V myosin Myo2 and Rab GTPase Sec4. These findings suggest that Sec15 play an important role in the polarised cell growth in *C. albicans*.

## Methods

### Strains, media and growth conditions

*C. albicans* strains used in this study are listed in Table 1. The strains were grown in either YPD (1% [w/v] yeast extract, 2% [w/v] peptone, 2% [w/v] glucose) or GMM medium (2% [w/v] glucose, 0.67% [w/v] yeast nitrogen base without amino acids). For *C. albicans* yeast growth, cells were grown at 30 °C. For hyphal growth, the media were supplemented with 10% serum and incubated at 37 °C. G1 cells were prepared by centrifugal elutriation as previously described^35^.

### Gene deletion

Gene deletion mutants were constructed by sequentially deleting the two copies of a gene from BWP17. For the deletion of *RSR1* gene, 500 bp of the 5′ UTR (AB) and 500 bp of the 3′ UTR (CD) of *RSR1* were sequentially cloned into the plasmid pGEM T-easy Vector (Promega, USA). Then the *HIS1* and *ARG4* gene sequence were inserted between AB and CD by a BamHI site respectively. These plasmids were used as templates for PCR amplification of *RSR1* gene deletion construct. To construct *SEC15* shut off strain controlled by the *MAL2* promoter, a *URA3* flipper cassette was constructed by flanking the 4.2 kb *URA3* flipper^36^ with the AB and CD DNA fragments corresponding to the 5′ and 3′ UTR of the *SEC15*. The *URA3* flipper cassette was used to disrupt one copy of *SEC15* from BWP17 as described previously^36^. The second copy of *SEC15* was placed under the control of *MAL2* promoter as described previously^37^. All strain genotypes were verified by colony PCR.

### Epitope tagging of proteins

To generate Exo84-GFP fusion protein under the control of its endogenous promoter, the C-terminal 600 bp of *EXO84* (orf19.135) coding sequence was amplified by PCR with *Kpn*I and *Xho*I sites added to 5′ and 3′ respectively, cleaved with *Kpn*I and *Xho*I, and cloned in frame at the *Kpn*I/*Xho*I sites of the plasmid pGFPutr^38^. The resulting plasmid was linearized by *EcoRV* at nt 2213 for chromosomal integration. To construct GFP-Exo70 fusion protein under the control of *MET3* promoter, the full length coding sequence of *EXO70* (orf19.6512) together with 500 bp of its 3′ UTR sequence was amplified by PCR with *Cla*I and *Pst*I sites added to 5′ and 3′ respectively, cleaved with *Cla*I and *Pst*I, and cloned in frame at the *Cla*I and *Pst*I sites of the plasmid pMET3-GFP-GAL4UTR^39^. The resulting plasmid was linearized by *Hind*III at a unique *Hind*III site in the 3′ UTR for chromosomal integration. To construct Sec5-GFP fusion protein under the control of its endogenous promoter, the C-terminal 2300 bp of *SEC5* (orf19.74) coding sequence was amplified by PCR with *Kpn*I and *Xho*I sites added to 5′ and 3′ respectively, cleaved with *Kpn*I and *Xho*I, and cloned in frame at the *Kpn*I/*Xho*I sites of the plasmid pGFPutr. The resulting plasmid was linearized by *EcoR*V at nt 742 for chromosomal integration. To construct Sec6-GFP fusion protein under the control of its endogenous promoter, the C-terminal 800 bp of *SEC6* (orf19.5463) coding sequence was amplified by PCR with *Kpn*I and *Xho*I sites added to 5′ and 3′ respectively, cleaved with *Kpn*I and *Xho*I, and cloned in frame at the *Kpn*I/*Xho*I sites of the plasmid pGFPutr. The resulting plasmid was linearized by *Hind*III at nt 1909 for chromosomal integration. To construct Sec10-GFP fusion protein under the control of its endogenous promoter, the full length coding sequence of *SEC10* (orf19.3086) coding sequence was amplified by PCR with *Kpn*I and *Xho*I sites added to 5′ and 3′ respectively, cleaved with *Kpn*I and *Xho*I, and cloned in frame at the *Kpn*I/*Xho*I sites of the plasmid pGFPutr. The resulting plasmid was linearized by *Nco*I at nt 453 for chromosomal integration. To construct Bem1-GFP fusion protein under the control of its endogenous promoter, the C-terminal 1500 bp of *BEM1* (orf19.4645) coding sequence was amplified by PCR with *Kpn*I and *Xho*I sites added to 5′ and 3′ respectively, cleaved with *Kpn*I and *Xho*I, and cloned in frame at the *Kpn*I/*Xho*I sites of the plasmid pGFPutr. The resulting plasmid was linearized by *Hind*III at nt 755 for chromosomal integration.

To construct RSR1^K16N^- MYC-*ARG4* fusion protein under the control of its endogenous promoter, the promoter sequence (1200 bp) together with the coding sequence of *RSR1* was amplified by PCR with *Kpn*I and *Xho*I sites added to 5′ and 3′ respectively, cleaved with *Kpn*I and *Xho*I, and cloned at the *Kpn*I/*Xho*I sites of the plasmid pMET3-6Myc-GAL4UTR^39^ to replace the *MET3* promoter, resulting plasmid pRSR1-6Myc. The plasmid pRSR1^K16N^-6Myc was generated using Quick Change Site-directed Mutagenesis Kit (Stratagene). Finally, plasmid pRSR1^K16N^-6Myc-*ARG4* was generated by replacing the *URA3* gene sequence with *ARG4* gene sequence using *Not*I and *Mlu*I sites. This plasmid was linearized by *EcoR*V at nt -921 in the promoter region for chromosomal integration. To construct Myo2- MYC-*ARG4* fusion protein under the control of its endogenous promoter, the C-terminal 686 bp of *MYO2* coding sequence was amplified by PCR with *Kpn*I and *Xho*I sites added to 5′ and 3′ respectively, cleaved with *Kpn*I and *Xho*I, and cloned at the *Kpn*I/*Xho*I sites of the plasmid pRSR1^K16N^ -6Myc -*ARG4* to replace RSR1 sequence. The resulting plasmid was linearized by *BamH*I at nt 4246 for chromosomal integration. To construct Bem1-HA fusion protein under the control of its endogenous promoter, the C-terminal 1500 bp of *BEM1* (orf19.4645) coding sequence was amplified by PCR with *Kpn*I and *Xho*I sites added to 5′ and 3′ respectively, cleaved with *Kpn*I and *Xho*I, and cloned at the *Kpn*I/*Xho*I sites of the plasmid pMET3-6HA-GAL4UTR^30^ to replace the *MET3* promoter. The resulting plasmid was linearized by *Hind*III at nt 755 for chromosomal integration.

### *In vitro* binding assay

For the binding assay between Sec15 and Rsr1, the full length coding sequence of Rsr1 was amplified from a cDNA library and fused to GST (GST-Rsr1) using the pGEX vector system (Amersham Biosciences). The GDP-locked form of Rsr1, GST-Rsr1^K16N^ was generated using Quick Change Site-directed Mutagenesis Kit (Stratagene). Three different fragments of Sec15 were amplified and subcloned into pET21-a (Novagen) to generate His-Sec15^1–300^ (amino acids 1 to 300), His-Sec15^1–549^ (amino acids 1 to 549) and His-Sec15^550–892^ (amino acids 550 to 892). The fusion proteins were purified from *Escherichia coli* according to the manufacturer’s protocol and used for *in vitro* binding assays. For a typical binding experiment, the GST-Rsr1^K16N^ fusion protein immobilized to glutathione-Sepharose beads (20 μl of beads) was incubated with 20 μg of purified His-Sec15^1–300^, His-Sec15^1–549^ and His-Sec15^550–892^ respectively in binding buffer (1× PBS buffer containing 1 mg/ml ovalbumin, 10 mM β-mercaptoethanol, and 0.1% IGPAL-30) for 1 hour at room temperature. The total volume of incubation mixture was 200 μl. After the resin was washed four times with the binding buffer excluding ovalbumin, bound GST fusion proteins were detected by Coomassie brilliant blue staining and associated His-tagged proteins were detected with anti-His antibody. Similar strategies were used for *in vitro* binding assays between Sec15 and Bem1 or Myo2.

### Co-immunoprecipitation

A culture of 20 ml was grown overnight in GMM. The culture was diluted to 200 ml the next day and grown for another 2 h. Cells were harvested and lysed in 1.5 volumes of HEPES lysis buffer (50 mM Hepes, pH 7.5, 1 mM EDTA, 5% glycerol, 0.05% Tween 20, 105 mM KCl) with complete protease inhibitor mix (Roche). Cells were broken by five rounds of 45-second beating at 5000 rpm in Tomy Micro Smash^TM^ MS-100 (Tomy Medico, Japan) with 1 minute incubation on ice between rounds. Cell lysate was clarified by spinning at 10,000 g twice. Antibody-conjugated beads (sc-8334 AC for GFP, sc-40 AC for Myc and sc-7392 AC for HA, Santa Cruz Biotechnology) were washed twice with 500 μl of lysis buffer and mixed with the clarified lysate and incubated at 4 °C for 2 h. After four times of wash with lysis buffer, the beads were boiled for 5 minutes with 1X loading buffer. Proteins were separated by SDS-PAGE and probed by Western Blot using appropriate antibodies (sc-8334 for GFP, sc-40 for Myc and sc-7392 for HA, Santa Cruz Biotechnology).

### Cell wall staining and fluorescence microscopy

Cell wall staining and fluorescence microscopy were performed as described by Pringle and co-workers^40^. A Leica DMR fluorescence microscope with 100× objective and a Hamamatsu digital camera interfaced with METAMORPH software (Universal Imaging) were used for imaging.

## Additional Information

**How to cite this article**: Guo, P. P. *et al.* Sec15 links bud site selection to polarised cell growth and exocytosis in *Candida albicans. Sci. Rep.* **6**, 26464; doi: 10.1038/srep26464 (2016).

## Supplementary Material

Supplementary Information

## Figures and Tables

**Table 1:** *Candida albicans* strains used in this study.

Strain	genotype	Source
BWP17	*ura3/ura3 his1*Δ*/his1*Δ *arg4*Δ*/arg4*Δ	41
AHU1	BWP17 *SEC15/sec15∆*::flp	This study
AHU2	BWP17 *sec15∆*::flp*/* P_MAL2_*-SEC15-HIS1*	This study
WYL41	BWP17 *SEC15/SEC15-GFP-URA3*	27
WYL31	BWP17 *SEC3/SEC3-GFP-URA3*	27
AHU3	BWP17 *SEC15/SEC15-GFP-URA3 RSR1/ RSR1*^*K16N*^*- MYC-ARG4*	This study
AHU4	BWP17 *RSR1/RSR1*^*K16N*^*- MYC-ARG4*	This study
AHU5	BWP17 *rsr1∆*::*ARG4/ rsr1∆*::*HIS1*	This study
AHU6	BWP17 *rsr1∆*::*ARG4/ rsr1∆*::*HIS1 SEC15/SEC15-GFP-URA3*	This study
AHU7	BWP17 *rsr1∆*::*ARG4/ rsr1∆*::*HIS1 SEC3/SEC3-GFP-URA3*	This study
AHU8	BWP17 *rsr1∆*::*ARG4/ rsr1∆*::*HIS1 EX070/EXO70-GFP-URA3*	This study
AHU9	BWP17 *rsr1∆*::*ARG4/ rsr1∆*::*HIS1 EXO84/EXO84-GFP-URA3*	This study
AHU10	BWP17 *rsr1∆*::*ARG4/ rsr1∆*::*HIS1 SEC5/SEC5-GFP-URA3*	This study
AHU11	BWP17 *rsr1∆*::*ARG4/ rsr1∆*::*HIS1 SEC6/SEC6-GFP-URA3*	This study
AHU12	BWP17 *rsr1∆*::*ARG4/ rsr1∆*::*HIS1 SEC10/SEC10-GFP-URA3*	This study
AHU13	BWP17 *EXO84/EXO84-GFP-URA3*	This study
AHU14	BWP17 *SEC5/SEC5-GFP-URA3*	This study
AHU15	BWP17 *SEC6/SEC6-GFP-URA3*	This study
AHU16	BWP17 *SEC10/SEC10-GFP-URA3*	This study
AHU17	BWP17 *SEC15/SEC15-GFP-URA3 BEM1/BEM1-HA-ARG4*	This study
AHU18	BWP17 *BEM1/BEM1-HA-ARG4*	This study
AHU19	BWP17 *sec15∆*::flp*/* P_MAL2_*-SEC15-HIS1 BEM1/BEM1-GFP-URA3*	This study
AHU20	BWP17 *SEC15/SEC15-GFP-URA3 MYO2/MYO2-MYC-ARG4*	This study
AHU21	BWP17 *MYO2/MYO2-MYC-ARG4*	This study
AHU22	BWP17 *EX070/EXO70-GFP-URA3*	This study
