# Supplementary material for: Sec15 links bud site selection to polarised cell growth and exocytosis in Candida albicans
Source: Sci Rep. 2016 May 26;6:26464. doi: 10.1038/srep26464 (PMC4881018; doi:10.1038/srep26464)
Supplement: Supplementary Information [file srep26464-s1.pdf]

Sec15 links bud site selection to polarized cell growth and exocytosis in *Candida albicans*

Pan Pan Guo, Jie Ying Au Yong, Yan Ming Wang and Chang Run Li

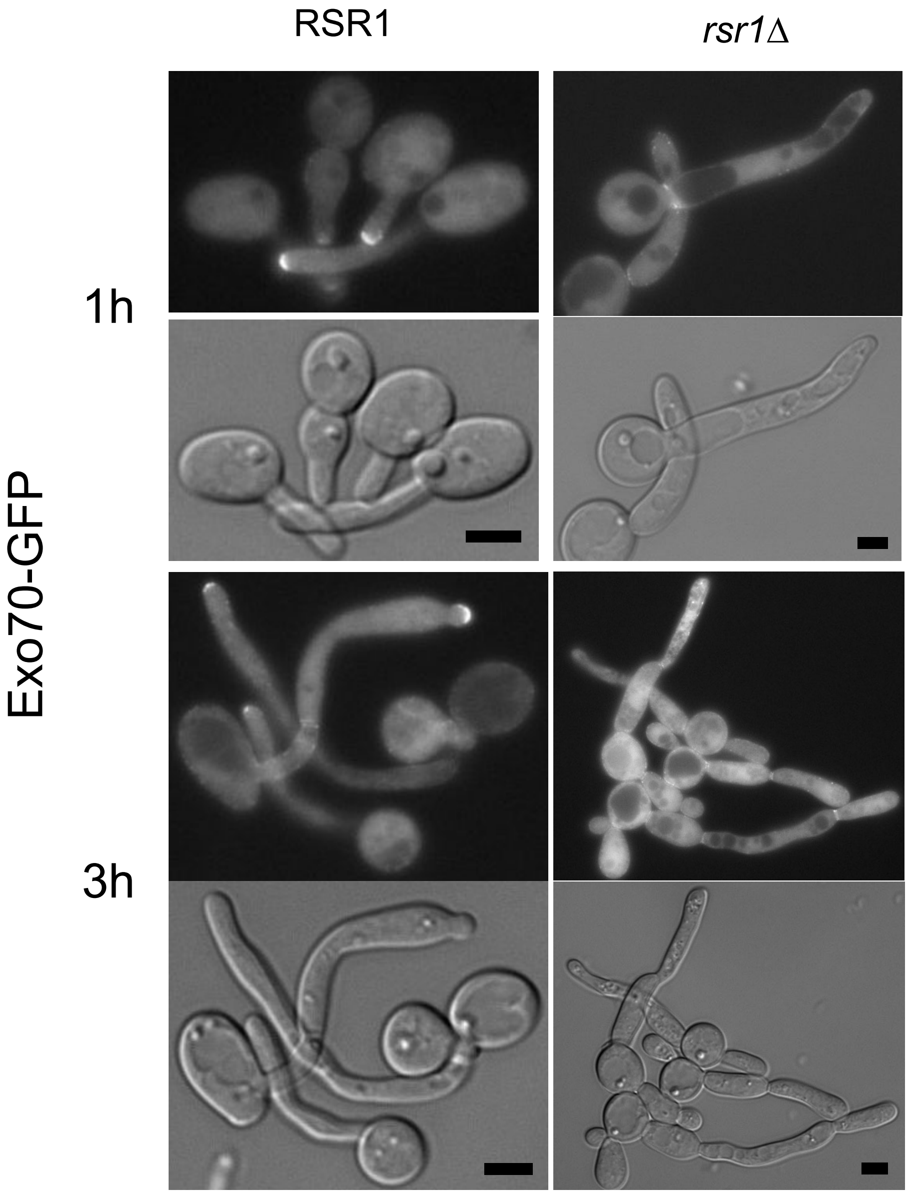

Figure S1

Exo70-GFP localization in wild type and *rsr1Δ* cells under hyphal growth condition. Bars, 5 μm.

Sec15 links bud site selection to polarized cell growth and exocytosis in *Candida albicans*

Pan Pan Guo, Jie Ying Au Yong, Yan Ming Wang and Chang Run Li

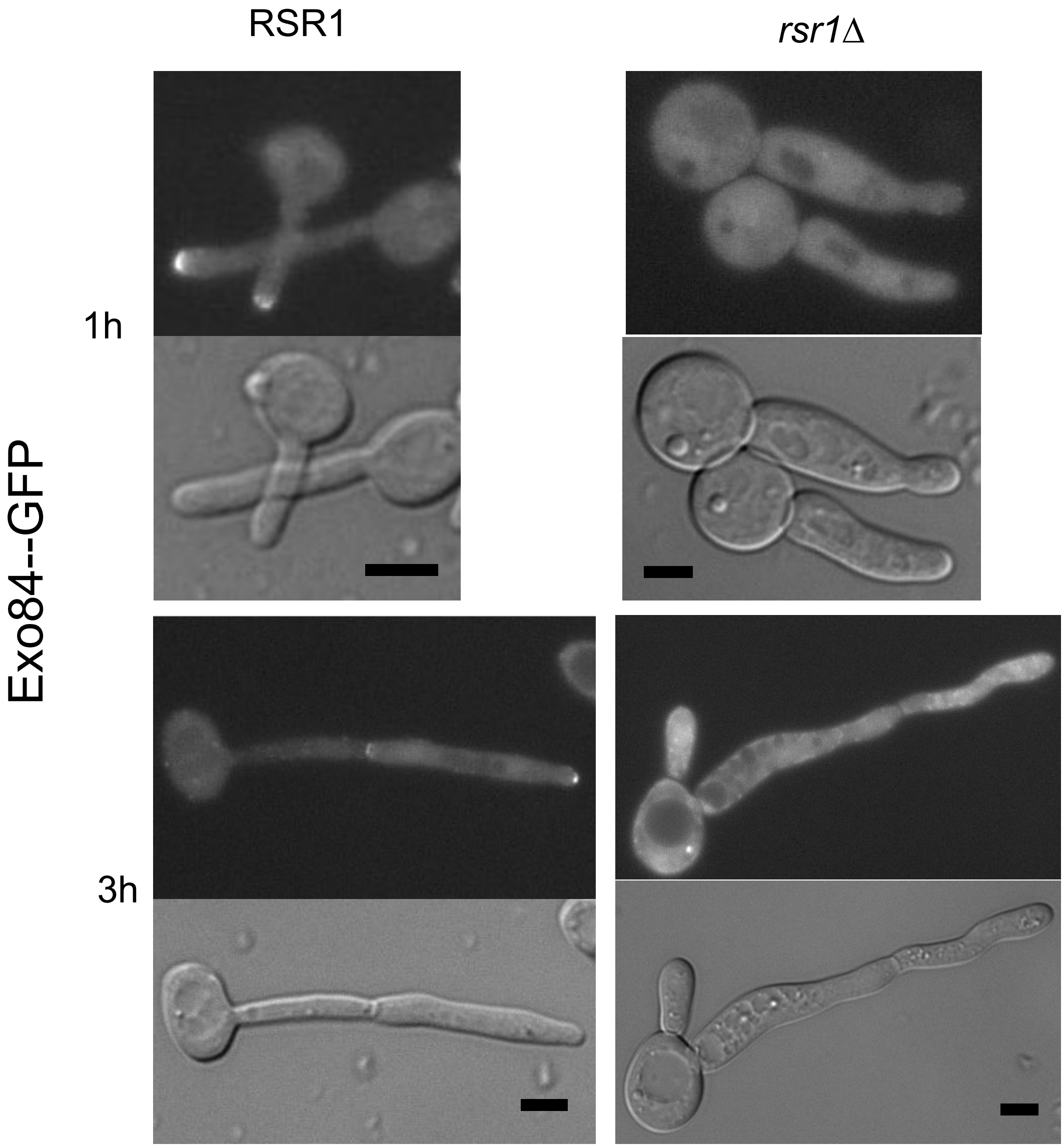

Figure S2

Exo84-GFP localization in wild type and *rsr1*Δ cells under hyphal growth condition. Bars, 5 μm.

Sec15 links bud site selection to polarized cell growth and exocytosis in *Candida albicans*

Pan Pan Guo, Jie Ying Au Yong, Yan Ming Wang and Chang Run Li

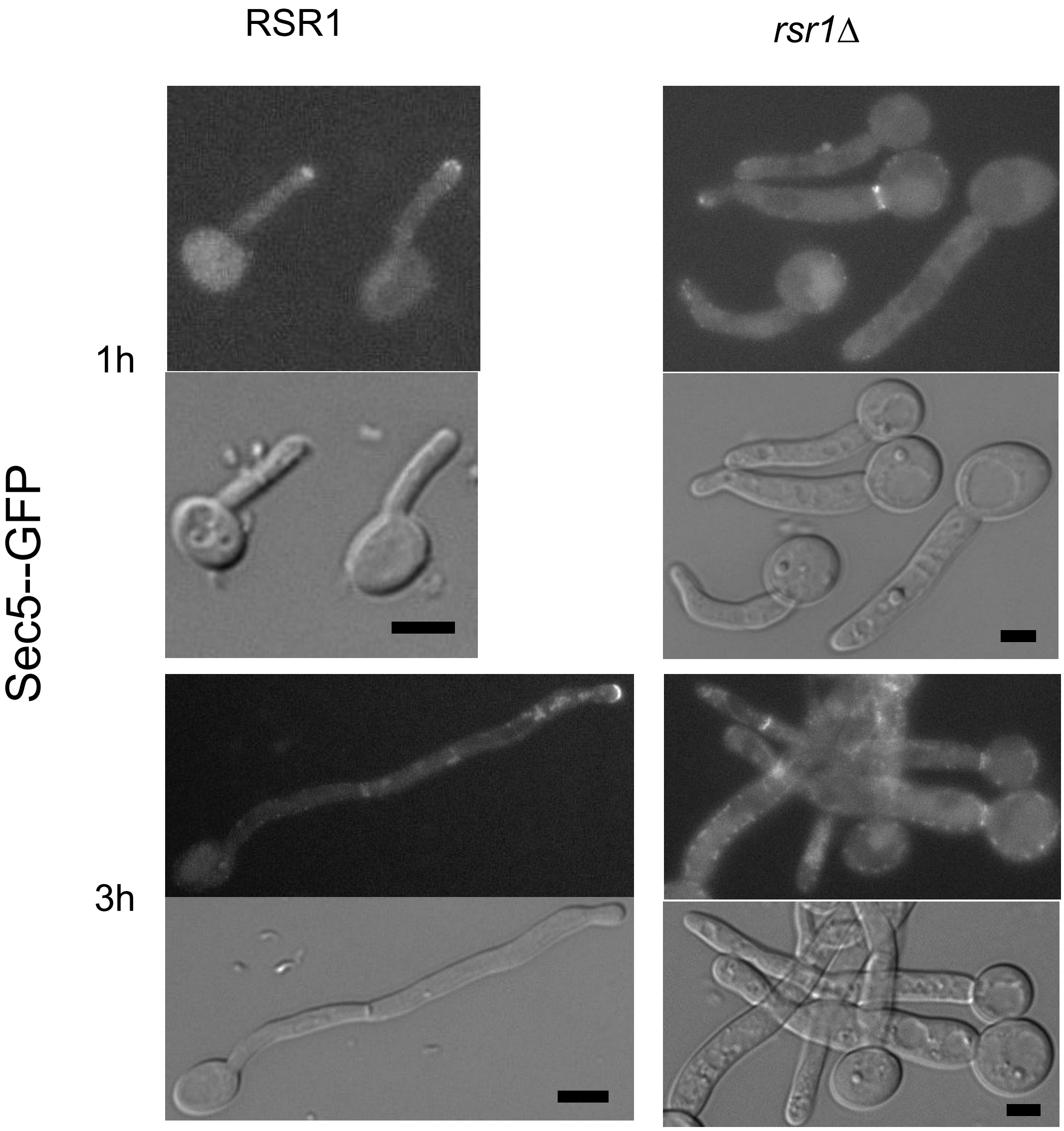

Figure S3

Sec5-GFP localization in wild type and *rsr1Δ* cells under hyphal growth condition. Bars, 5 μm.

Sec15 links bud site selection to polarized cell growth and exocytosis in *Candida albicans*

Pan Pan Guo, Jie Ying Au Yong, Yan Ming Wang and Chang Run Li

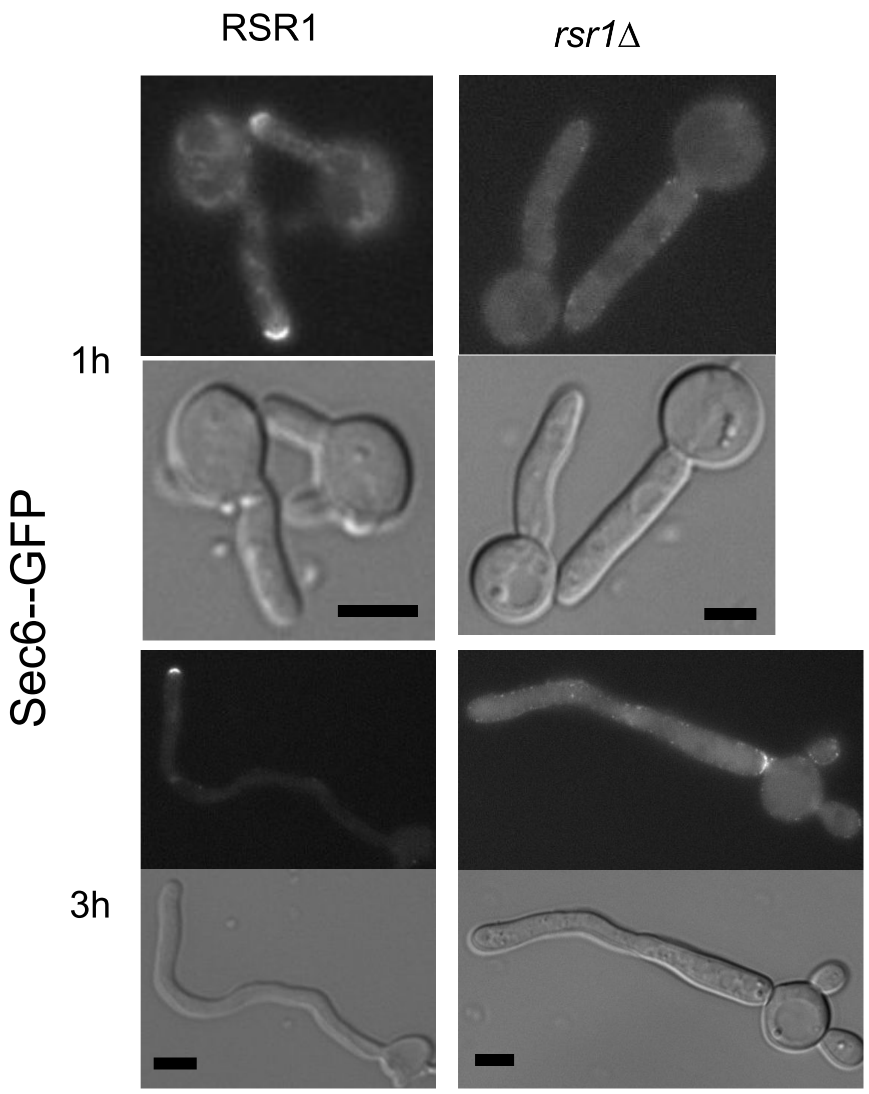

Figure S4

Sec6-GFP localization in wild type and *rsr1*Δ cells under hyphal growth condition. Bars, 5 μm.

Sec15 links bud site selection to polarized cell growth and exocytosis in *Candida albicans*

Pan Pan Guo, Jie Ying Au Yong, Yan Ming Wang and Chang Run Li

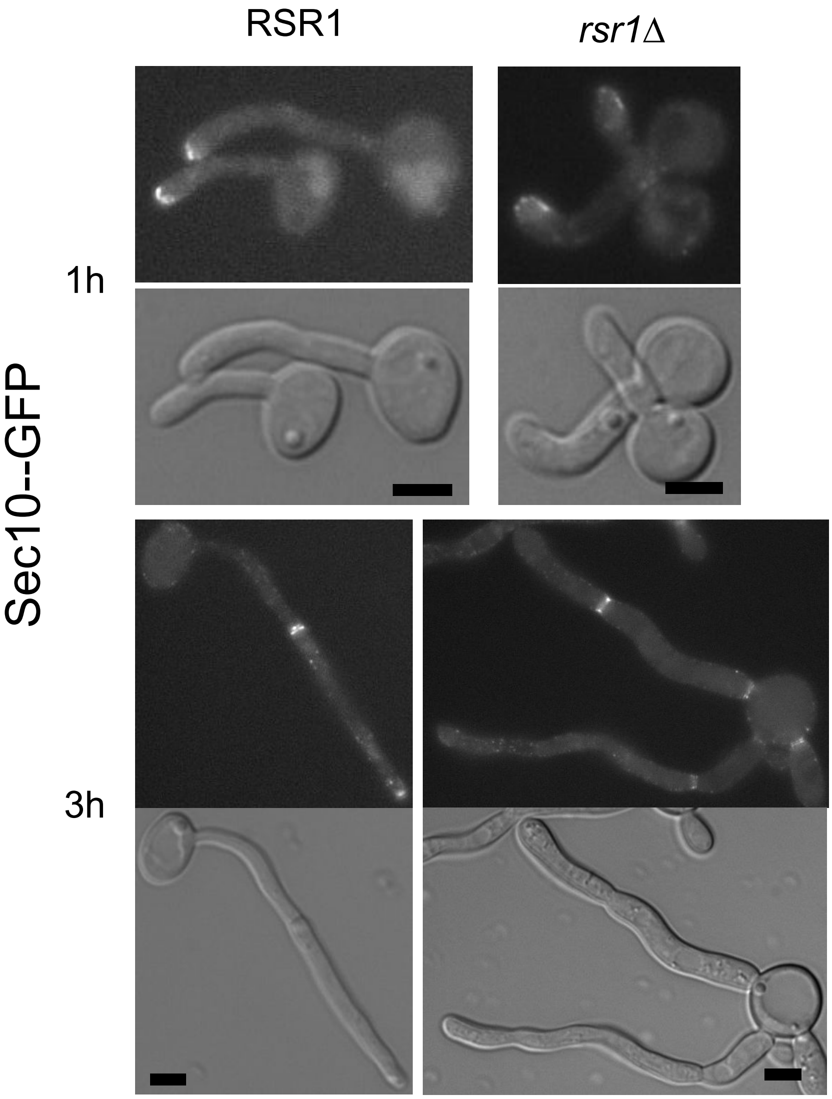

Figure S5

Sec10-GFP localization in wild type and *rsr1*Δ cells under hyphal growth condition. Bars, 5 μm.
